# Pancreaticoduodenectomy after Roux-en-Y gastric bypass and novel endoscopic ultrasound-directed transgastric ERCP procedure

**DOI:** 10.1093/jscr/rjae762

**Published:** 2024-12-02

**Authors:** Carolina Orsi, Tyler Davis, Paige Moudy, Hishaam Ismael

**Affiliations:** Department of Graduate Medical Education, General Surgery, The University of Texas at Tyler Health Science Center, 11937 US HWY 271, Tyler, TX 75708, United States; Department of Graduate Medical Education, General Surgery, The University of Texas at Tyler Health Science Center, 11937 US HWY 271, Tyler, TX 75708, United States; Department of Graduate Medical Education, General Surgery, The University of Texas at Tyler Health Science Center, 11937 US HWY 271, Tyler, TX 75708, United States; Department of Surgery, The University of Texas Health Science Center at Tyler, 11937 US HWY 271, Tyler, TX 75708, United States

**Keywords:** pancreaticoduodenectomy, gastric bypass, ERCP, EDGE, adenocarcinoma

## Abstract

Performing a pancreaticoduodenectomy (PD) in patients having undergone a Roux-en-Y gastric bypass (RNYGB) poses a significant surgical challenge. We present a patient with a history of RNYGB and endoscopic ultrasound-directed transgastric ERCP (EDGE) procedure who underwent a successful PD. This 77-year-old female with history of open RNYBG presented with resectable pancreatic adenocarcinoma. A preoperative EDGE procedure was required for biliary decompression. A PD was performed by removing the entire biliopancreatic limb for oncologic resection. The reconstructive technique here involved utilizing the old common channel for the hepaticojejunostomy, pancreaticojejunostomy, and remnant gastrojejunostomy. The case also included Axios stent placement during a preoperative EDGE procedure. This case describes the first reported successful PD in a patient with prior RNYGB and EDGE procedure. Although the optimal technique for this clinical scenario remains unestablished, this unique case contributes to the literature by demonstrating an effective approach for practicing surgeons.

## Introduction

Pancreatic cancer is characterized as one of the most lethal and aggressive malignancies [1], with a 5-year survival rate of ~12% [2]. Modifiable risk factors include tobacco use, diet type, and obesity [1, 3]. Regarding obesity, the associated abnormal metabolism, insulin resistance, and glucose intolerance closely contributes to the correlation between obesity and the development of pancreatic cancer [4]. One of the most common surgical treatment strategies for obesity includes the Roux-En-Y gastric bypass (RNYGB). With the growing obesity epidemic, it is reasonable to predict that patients may develop pancreatic cancer in the setting of a previous bariatric procedure. Therefore, it is helpful to understand the options that exist when performing surgical resections for pancreatic cancers in bariatric patients.

Here, we describe a patient with previous RNYGB who underwent a successful pancreaticoduodenectomy (PD) for pancreatic adenocarcinoma. The preoperative clinical course involved endoscopic ultrasound directed transgastric endoscopic retrograde cholangiopancreatography (EDGE) for biliary decompression and tissue diagnosis. The intraoperative approach involved complete resection of the biliopancreatic (BP) limb, creation of the pancreaticojejunostomy (PJ), and hepaticojejunostomy (HJ) with the common channel, and construction of a gastrojejunostomy (GJ) to the remnant stomach. We aim for this surgical technique to offer a successful and effective option for surgeons encountering PD reconstruction in patients with RNYGB.

## Case report

**Figure 1:**
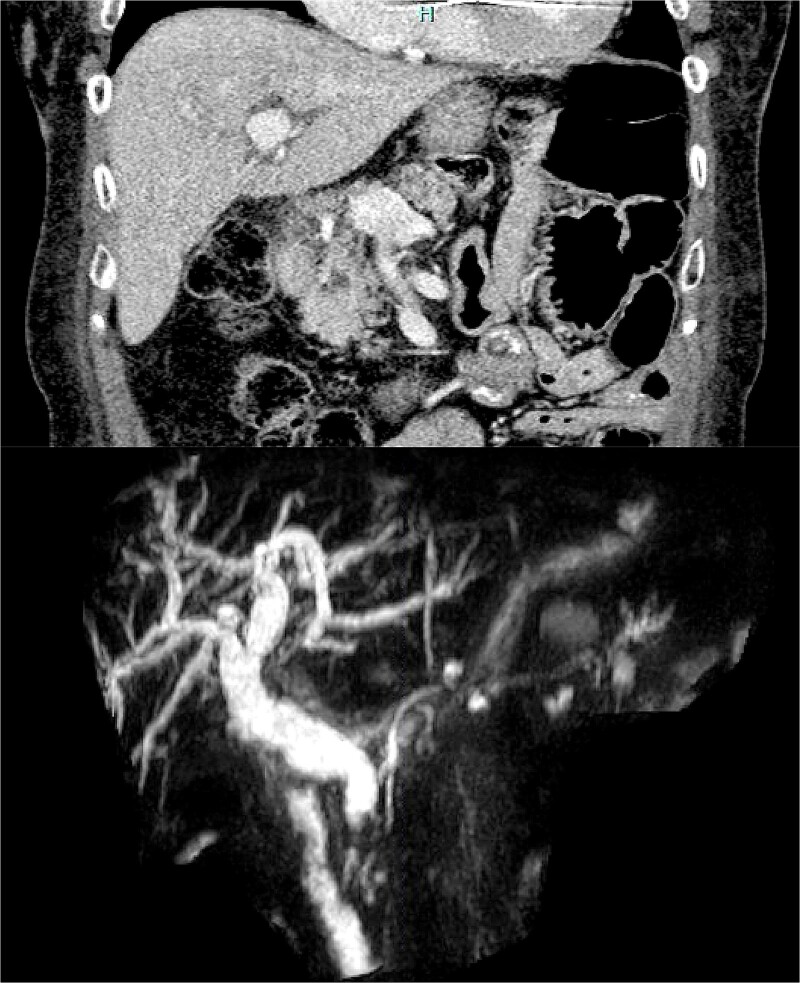
(a) CT scan image of the cystic lesion within the head of the pancreas. (b) MRCP image of intrahepatic and extrahepatic bile duct dilation with abrupt change in caliber at the distal CBD.

This patient was a 77-year-old female with a past surgical history including open RNYGB and open cholecystectomy. She initially presented with abdominal pain and transaminitis, although her total bilirubin was normal. A CT scan revealed intrahepatic and extrahepatic bile duct dilation with a cystic lesion within the pancreatic head (Fig. 1a). Magnetic resonance cholangiopancreatography (MRCP) confirmed a high-grade stricture of the distal common bile duct (CBD), concerning for malignancy (Fig. 1b)*.*

**Figure 2:**
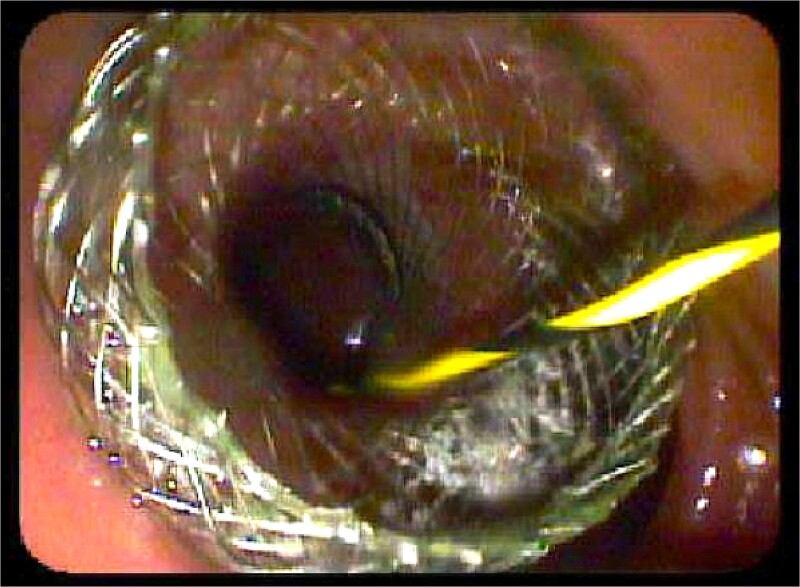
Endoscopic view of the Axios stent during preoperative EDGE procedure.

During her hospital course, her total bilirubin gradually increased. Therefore, the EDGE procedure was undertaken for diagnostic utility and therapeutic biliary decompression. This involved placing a 20-mm axios stent between the Roux-limb and remnant stomach under endoscopic ultrasound guidance (Fig. 2)*.* The stent was unable to be placed between the gastric pouch and remnant stomach per usual technique due to the gastric pouch being supradiaphragmatic. Endoscopic ultrasound (EUS), fine-needle aspiration (FNA), and ERCP were then completed 2 weeks later by endoscopically traversing the Axios stent to gain periampullary access. Sphincterotomy and CBD stenting were performed.

After multidisciplinary discussions, a PD was planned due to the tumor’s resectability and lack of vascular involvement. Intraoperatively, an extensive lysis of adhesions was executed. Once her anatomy was mobilized, a retrocolic, retrogastric RNY configuration was found with a dilated BP limb. Fortunately, no carcinomatosis or liver metastases were seen.

At this point, the standard steps were utilized to proceed with the PD. The entirety of the BP limb was then resected by performing small bowel resection at the jejuno-jejunostomy. For reconstruction, an end-to-side PJ with the duct-to-mucosa, Blumgart [5] surgical technique was performed by utilizing the old common channel. A single-layer HJ was then created in the standard fashion. Following this, due to the close proximity of the antrectomy staple line to the Axios stent, a standard two-layer, handsewn GJ was created to the gastric remnant in isoperistaltic orientation along the greater curve. To reestablish bowel continuity, the Roux limb distal to the Axios stent was anastomosed to the distal common channel.

Post-operatively, the nasogastric tube was removed on postoperative day (POD) 3. The patient’s diet was advanced toward a bariatric, regular diet on POD 5. She was discharged on POD 6. After discharge, her only complication involved pain control. The Axios stent was removed 8 months later.

## Discussion

Those with pancreatic cancers involving the pancreatic head that are deemed resectable require a PD, often known as the Whipple procedure [5]. This procedure is incredibly complex, requiring resection of the duodenum, proximal jejunum, CBD, gallbladder, pancreatic head, and distal stomach, followed by reconstruction [6]. For a RNYGB, a small gastric pouch is created, the proximal jejunum divided, and a Roux-en-Y configuration is constructed [7]. This altered bariatric anatomy poses a challenging surgical scenario in patients requiring a PD, with only 26 specific cases documented in the literature previously [8, 9]. Therefore, it is vital to understand a patient’s anatomy to effectively plan and optimize outcomes in those requiring the PD after RNYGB.

**Figure 3:**
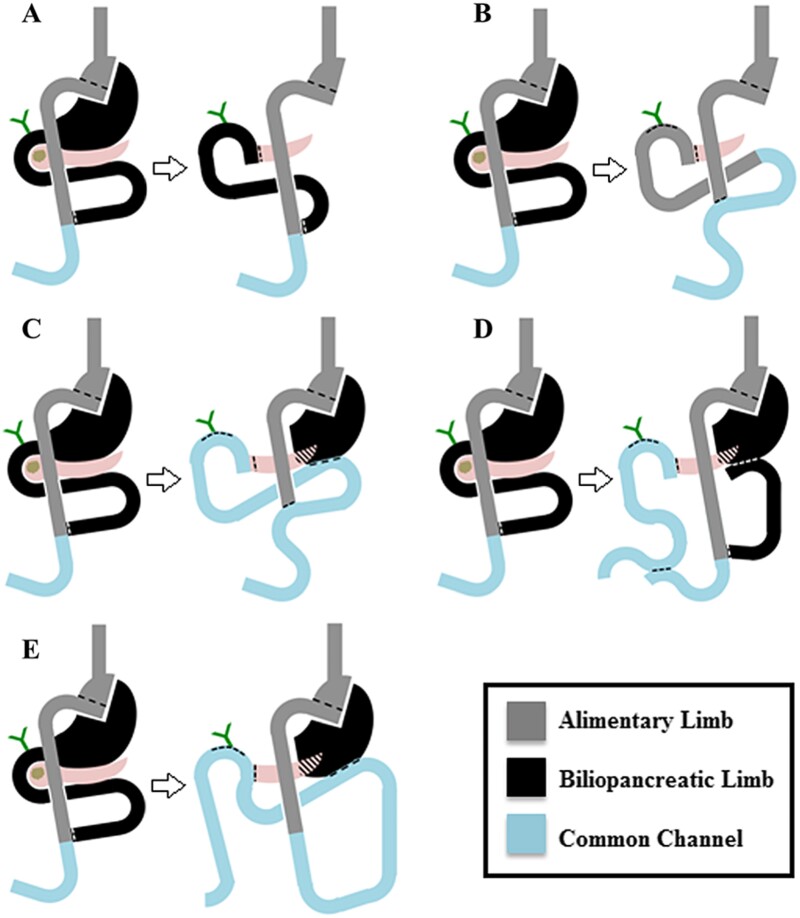
Illustrations of a variety of techniques presented by Morano *et al.* [8] involving a pancreaticoduodenectomy following RNYGB.

Morano *et al.* illustrates a variety of surgical techniques for PD after RNYGB (Fig. 3) [8]. In our case, the retrogastric, retrocolic position of the gastric pouch was an important anatomical consideration. Given the dilated BP limb and dense adhesions encountered, the technique employed closely resembles that of option C as shown in Fig. 1 [8]. By resecting the BP limb completely, HJ and PJ reconstruction could be performed in standard fashion after removal of the specimen.

The EDGE procedure is a novel endoscopic procedure. It is an ultrasound-guided technique that involves accessing the remnant stomach in order to gain transpapillary position for ERCP interventions in those with previous RNYGB [10]. Only a handful of reports are currently in the literature regarding its perioperative use in those with pancreatic cancers. In this case, it added to the complexity of this patient’s anatomy and intra-operative decision making.

The axios stent placed during preoperative EDGE procedure inevitably created a fixed connection and local inflammation between the Roux limb and gastric remnant, deeming it difficult for resection. The axios stent was also in close proximity to the staple line of the antrectomy, which potentially would jeopardize the integrity of an anastomosis. Therefore, the GJ was created in standard fashion along the greater curvature of the remnant stomach instead of at the antrectomy staple line. By doing so, a strong and widely patent GJ allowed the gastric remnant to stay in place with the Axios stent in stable position with its connection to the Roux limb and upstream gastric pouch.

In summary, this case represents the first reported surgical scenario where a patient with previous RNYGB and preoperative EGDE procedure successfully underwent a complex PD with innovative surgical reconstruction. Although a case report limits the universality of the approach described, we trust that this technique proves valuable and contributes to the existing literature a relevant option to surgeons for patients requiring PD after RNYGB.
